# Prussian Blue Analogue-Derived Fe-Doped CoS_2_ Nanoparticles Confined in Bayberry-like N-Doped Carbon Spheres as Anodes for Sodium-Ion Batteries

**DOI:** 10.3390/polym15061496

**Published:** 2023-03-17

**Authors:** Jiajia Hu, Cheng Liu, Chen Cai, Qianqian Sun, Mixue Lu, Zhujun Yao, Yefeng Yang

**Affiliations:** School of Materials Science and Engineering, Zhejiang Sci-Tech University, Hangzhou 310018, China

**Keywords:** sodium-ion batteries, anode, Fe-doped CoS_2_, coordination polymers, Prussian blue analogue

## Abstract

Obvious volume change and the dissolution of polysulfide as well as sluggish kinetics are serious issues for the development of high performance metal sulfide anodes for sodium-ion batteries (SIBs), which usually result in fast capacity fading during continuous sodiation and desodiation processes. In this work, by utilizing a Prussian blue analogue as functional precursors, small Fe-doped CoS_2_ nanoparticles spatially confined in N-doped carbon spheres with rich porosity were synthesized through facile successive precipitation, carbonization, and sulfurization processes, leading to the formation of bayberry-like Fe-doped CoS_2_/N-doped carbon spheres (Fe-CoS_2_/NC). By introducing a suitable amount of FeCl_3_ in the starting materials, the optimal Fe-CoS_2_/NC hybrid spheres with the designed composition and pore structure exhibited superior cycling stability (621 mA h g^−1^ after 400 cycles at 1 A g^−1^) and improved the rate capability (493 mA h g^−1^ at 5 A g^−1^). This work provides a new avenue for the rational design and synthesis of high performance metal sulfide-based anode materials toward SIBs.

## 1. Introduction

With the ever-growing demand for energy storage and conversion systems to address the great challenges of global environmental pollution and climate change, it has become imperative to develop efficient and renewable rechargeable batteries, which play an indispensable role in modern human society [1,2,3]. In recent years, sodium-ion batteries (SIBs) have stood out as one type of promising energy storage devices because of the low cost and natural abundance of sodium resource, suitable redox potential as well as their similar physical and chemical properties to lithium-ion batteries (LIBs) [4,5,6]. As we know, the performances of SIB devices are mainly dependent on their anodes [7,8]. Hence, many efforts have been devoted to exploring suitable anode materials such as carbonaceous materials [9,10], metal alloys [11,12], metal sulfides [13,14,15,16], and selenides [17,18]. Among them, cobalt disulfide (CoS_2_) has attracted considerable research attention due to the high theoretical capacity (870 mA h g^−1^), environmental benignity, high electrochemical reversibility, and good compatibility with carbon [19,20,21]. However, the obvious volume change and dissolution of polysulfide during sodiation and desodiation, and the sluggish kinetics induced by the larger ionic radius of Na^+^ compared with that of Li^+^, usually result in an inferior cycling stability and rate performance, which greatly restrict the practical application of CoS_2_ in SIBs [22,23].

To solve these problems, several effective strategies including hetero-atom doping [24], carbon integration [25], and construction of hybrid nanostructures [26] have been adopted by various groups to achieve the goal of improving the sodium storage properties of CoS_2_. For instance, Xu et al. reported the fabrication of Ni-doped CoS_2_ embedded in N, P, Co-doped biomass-derived carbon spheres, exhibiting a capacity of 302 mA h g^−1^ at 1 A g^−1^ after 50 cycles [27]. In another work, Dong and co-workers illustrated the preparation of a Prussian blue analogue (PBA) Mn_3_[Co(CN_6_)]_2_·9H_2_O derived bimetallic sulfide MnS-CoS_2_-NC@NC with a hollow nanocubic structure through a simple one-step calcination, delivering a capacity of 609 and 561 mA h g^−1^ at 0.2 and 5 A g^−1^, respectively [28]. More recently, Feng’s group reported the preparation of metal–organic framework (MOF) derived CoS_2_ nanoparticles embedded in N-doped carbon on Ti_3_C_2_T_x_ MXene nanosheets, achieving a capacity of 355 mA h g^−1^ at 5 A g^−1^ after 5000 cycles [29]. Zhao et al. reported the fabrication of CoS_2_ nanoparticles on three-dimensional N-doped carbon, which displayed a capacity of 341 mA h g^−1^ after 1000 cycles at 1 A g^−1^ [30]. Introducing metal atoms into transition metal compounds is an effective strategy to improve the electrochemical performance. The doping of metal elements can regulate the electronic structure, form a synergistic effect, increase the active sites, improve the conductivity and stability, and also effectively promote the dynamics of electrode materials [31,32]. Fe^3+^ is a promising dopant ion with a low price, is non-toxic, and an abundant natural resource [33]. Li et al. designed a flower-like Fe-doped CoP on a carbon membrane, and the hierarchical Fe-doped CoP directly grown on the carbon membrane increased the active sites for the intercalation of Na^+^, exhibiting a capacity of 515 mA h g^−1^ at 1 A g^−1^ after 100 cycles [34]. Although great progress has been made in the rational design and utilization of CoS_2_-based anode materials for SIBs, the pursuit of anode materials with high capacity and stability is still a key challenge for the development of full battery technology. 

Herein, we report on the fabrication of a PBA-derived bayberry-like unique hybrid nanostructure comprising Fe-doped CoS_2_ nanoparticles confined in N-doped carbon spheres (labeled as Fe-CoS_2_/NC) through a combined method of precipitation, carbonization, and sulfurization. As an emerging class of coordination frameworks assembled by certain metal ions and ligands, PBA has been considered as versatile functional precursors for constructing a variety of transition metal sulfides with carbon modification due to the adjustable component, controllable size, and unique structure [35,36,37]. It should be mentioned that the introduced Pluronic F127 and as-formed Zn species at high temperature can be readily removed during the carbonization process, leading to the creation of numerous mesopores within the carbon spheres. Furthermore, the size of the Fe-CoS_2_/NC hybrid spheres can be tuned by introducing a suitable amount of FeCl_3_ in the starting materials, and as expected, the incorporated Fe^3+^ ions in the CoS_2_ matrix can act as additional active sites and contribute to the total capacity. Moreover, the exterior N-doped carbon spheres with rich porosity derived from the carbonization of PBA can not only boost the electron and ion transport, but also effectively restrain the volume variation during sodiation and desodiation, leading to the improved rate and cycling performance. As a result, the optimal anode of Fe-CoS_2_/NC hybrid spheres delivered a high capacity of 621 mA h g^−1^ after 400 cycles at 1 A g^−1^ and exhibited an elevated rate capability with a capacity of 493 mA h g^−1^ at 5 A g^−1^.

## 2. Materials and Methods

### 2.1. Synthesis of Fe-CoS_2_/NC Spheres

Typically, 1.5 mmol of Zn(CH_3_COO)_2_·2H_2_O, 1.8 g of polyvinyl pyrrolidone (PVP), 1 g of Pluronic F127 and a certain amount (*x* = 0.1, 0.2, 0.3 and 0.4 mmol) of FeCl_3_ were successively mixed in 50 mL of deionized water with magnetic stirring to obtain a clear and transparent solution. Subsequently, 50 mL of aqueous solution containing 1 mmol of K_3_[Co(CN)_6_] was slowly added into the above solution under stirring for 30 min. Afterward, the resulting solution was further aged for 24 h at room temperature and the collected yellowish precipitate was rinsed with deionized water and absolute ethanol and then dried at 80 °C overnight under vacuum, which was denoted as FeZnCo-PBA-1, FeZnCo-PBA-2, FeZnCo-PBA-3, and FeZnCo-PBA-4 for *x* equals 0.1, 0.2, 0.3, and 0.4, respectively. Next, the as-prepared FeZnCo-PBA samples were further annealed at 800 °C for 2 h under the protection of a N_2_ flow, and then subjected to sulfurization by excess sulfur powder at 500 °C for 3 h, leading to the formation of the desired composites of the Fe-CoS_2_/NC-1, Fe-CoS_2_/NC-2, Fe-CoS_2_/NC-3 and Fe-CoS_2_/NC-4 hybrid spheres, respectively. For comparison, the sample of the pristine CoS_2_/NC spheres was also synthesized under a similar reaction condition without the introduction of FeCl_3_ in the starting materials.

### 2.2. Material Characterization

The detailed morphology and particle size of the samples were characterized by field-emission scanning electron microscopy (SEM, Zeiss Ultra 55, Oberkochen, Germany) and high-resolution transmission electron microscopy (HRTEM, FEI TF20, Hillsboro, America). The phase structure of the samples was investigated by X-ray diffraction (XRD) on a Bruker D8 Advance X-ray diffractometer, Germany. The surface property of the samples was analyzed using X-ray photoelectron spectroscopy (XPS, Thermo Scientific K-Alpha, Waltham, America) with Al K_α_ radiation. The Brunauer–Emmett–Teller (BET) specific surface area of the samples was tested by N_2_ adsorption–desorption isothermal curves at 77 K using a BSD-PS1 instrument, and the Raman spectra were investigated using a Raman microscope (Renishaw in Via-Reflex, Gloucestershire, Britain) with an incident laser wavelength of 532 nm.

### 2.3. Electrochemical Measurements

For the electrochemical measurement, the CR2025-type coin cells were packed in a glovebox by using Na foil as the counter electrode, and sodium fluorophosphate (NaPF_6_, 1.0 M) dissolved in diethylene glycol dimethyl ether (DIGLYME) as the electrolyte, and glass fiber (grade GF/D) as the separator, respectively. The working electrodes were fabricated by mixing active materials (80 wt.%), super-P (10 wt.%), and polyvinylidene fluoride (PVDF. 10 wt.%) in a suitable amount of *N*-methyl-2-pyrrolidone (NMP) solvent, casting the resulting slurry onto a piece of Cu foil by a doctor-blade method, and drying at 80 °C for 12 h. Basically, the Cu foil was punched into a circle plate with a diameter of about 1.2 cm, and the area weight of active materials on the foil was about 1.5 mg cm^−2^. Cyclic voltammetry (CV) and electrochemical impedance spectroscopy (EIS) measurements were conducted on an electrochemical workstation of CHI 760E and Autolab PGSTAT 302 N, while the galvanostatic charge and discharge (GCD) tests were conducted on LAND CT2001A and Neware multi-channel battery testing systems from 0.01 to 3 V.

## 3. Results

**Figure 1:**
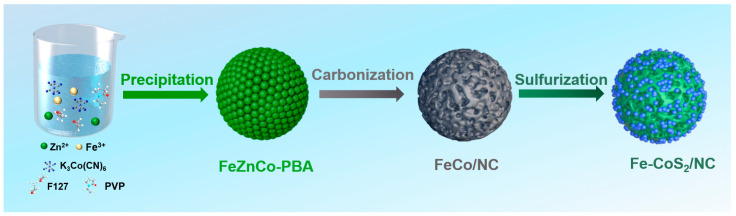
Schematic diagram of the fabrication process for the Fe-CoS_2_/NC spheres.

In this study, the fabrication process for the targeted Fe-CoS_2_/NC hybrid spheres consists of three major steps, as schematically indicated in Figure 1. First, the spherical FeZnCo-PBA particles as the precursor were prepared by a facile co-precipitation method at room temperature through the reaction of Zn^2+^, Fe^3+^, and [Co(CN)_6_]^3−^ in an aqueous environment under the assistance of PVP and Pluronic F127. During this process, PVP could effectively reduce the nucleation rate of FeZnCo-PBA spheres through the coordination of amide groups with transition metal ions and then adsorb them on the surface of FeZnCo-PBA spheres [38,39], while Pluronic F127, as the well-known soft template, could benefit the formation of mesopores within the carbon network through its facile decomposition at a high carbonization temperature [40,41,42]. In the subsequent step, FeCo/NC hybrid spheres were produced as expected through a high temperature carbonization treatment at 800 °C in an inert atmosphere, in which the transition metal ions were reduced to FeCo alloy nanoparticles while the adsorbed PVP and CN^−^ participating in the coordination reaction were simultaneously converted into an N-doped carbon skeleton. Furthermore, Pluronic F127 as well as the Zn species as-formed under the reductive carbon atmosphere could be removed at the high temperature. As shown in Figure S1, no signal arising from Zn could be detected, leading to the creation of abundant ordered mesopores within the carbon skeleton [43]. Eventually, the as-fabricated FeCo/NC hybrid spheres were subjected to a gas sulfurization process at 500 °C, and bayberry-like Fe-CoS_2_/NC hybrid spheres could be successfully obtained. It is noteworthy that by simplifying tuning the amount of FeCl_3_ added in the starting materials, a series of Fe-CoS_2_/NC hybrid spheres labeled as Fe-CoS_2_/NC-1, Fe-CoS_2_/NC-2, Fe-CoS_2_/NC-3, and Fe-CoS_2_/NC-4 could be achieved from the multi-step reactions. Considering the superior sodium storage properties of Fe-CoS_2_/NC-3, in this context, we will focus on this sample for further investigation.

**Figure 2:**
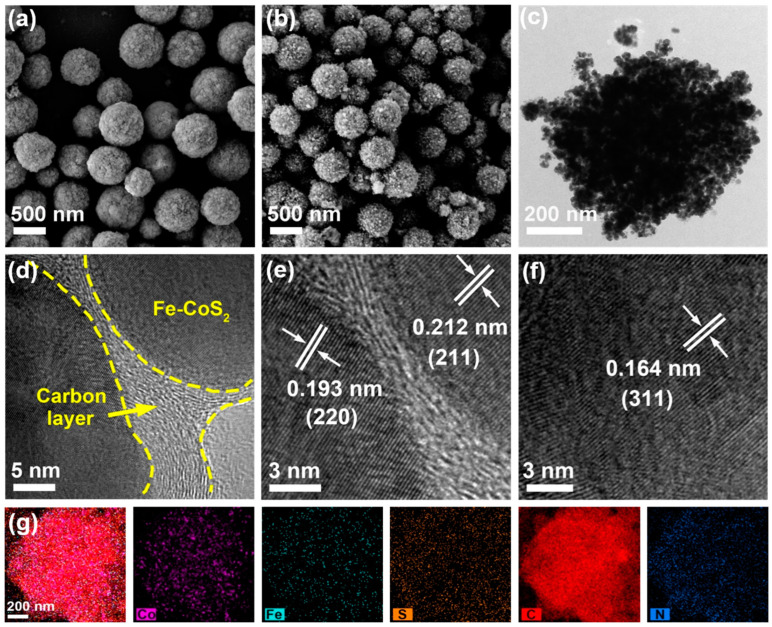
Typical SEM images of the (**a**) FeZnCo-PBA-3 precursor spheres and (**b**) Fe-CoS_2_/NC-3 hybrid spheres, (**c**) TEM image of an individual Fe-CoS_2_/NC-3 hybrid sphere, (**d**–**f**) HRTEM images, and (**g**) EDS element mapping analysis of Fe-CoS_2_/NC-3.

The detailed morphology and microstructure of the as-prepared samples were revealed by SEM and TEM. Figure 2a presents the SEM image of the FeZnCo-PBA-3 precursor spheres, showing an average size of *ca.* 572 nm with a relatively smooth surface. After being subjected to carbonization and sulfurization, the spherical structure of the FeZnCo-PBA-3 precursor was almost maintained by the bayberry-like Fe-CoS_2_/NC-3 spheres with a very rough surface and a reduced average size of *ca.* 450 nm, and a large number of small nanoparticles were found to decorate the surface of these Fe-CoS_2_/NC-3 spheres, as displayed in Figure 2b. Figure 2c displays a representative TEM image of an individual Fe-CoS_2_/NC-3 sphere with rich porosity, which was assembled from small dense nanoparticles possessing an average size of *ca.* 25 nm, further confirming the formation of a bayberry-like structure for the hybrid sphere. High-magnification TEM characterization, as shown in Figure 2d, indicated that these small crystalline nanoparticles were separately coated by a thin layer of carbon with a thickness of a few nanometers, which could contribute significantly to the improved electrical conductivity and reinforced structural integrity of the composite. More detailed HRTEM measurements at different regions (Figure 2e,f) showed several well-resolved lattice fringes with *d*-spacings of 0.212, 0.193, and 0.164 nm, corresponding well to the (211), (220), and (311) crystalline planes of the cubic CoS_2_ (JCPDS. No. 89-3056) [44], respectively. In addition, the elemental mapping analysis by EDS (Figure 2g) clearly indicates that the Co, Fe, and S elements were discretely located in the core part of the composite structure while the C and N elements nearly overlapped and were detected in the whole structure, implying that the Fe-doped CoS_2_ nanoparticles are intimately enveloped by a layer of the N-doped carbon skeleton to form a hybrid composite structure.

**Figure 3:**
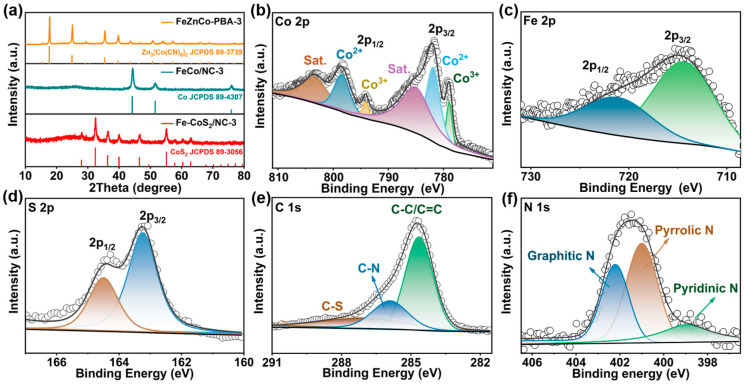
(**a**) XRD patterns of the FeZnCo-PBA-3, FeCo/NC-3 and Fe-CoS_2_/NC-3 spheres, (**b**–**f**) XPS spectra of the Fe-CoS_2_/NC-3 spheres: (**b**) Co 2p, (**c**) Fe 2p, (**d**) S 2p, (**e**) C 1s, and (**f**) N 1s, respectively.

To obtain more information about the structure and chemical composition of the samples, XRD and XPS tests were conducted, with the results presented in Figure 3. Figure 3a displays the XRD patterns of the FeZnCo-PBA-3, FeCo/NC-3, and Fe-CoS_2_/NC-3 spheres, indicating the structural evolution of the samples during the formation reaction. Specifically, all the characteristic peaks of the FeZnCo-PBA-3 sample were in good agreement with the cubic Zn_3_[Co(CN)_6_]_2_ phase (JCPDS No. 89-3739) [45]. After the carbonization treatment, we could see that the diffraction peaks of the FeCo/NC-3 sample mainly appeared at 44.3°, 51.5°, and 76.0° could be well-indexed to the (111), (200), and (220) planes of the cubic Co phase (JCPDS No. 89-4307) along with a weak and wide peak at about 26° [38,46], respectively, which was ascribed to the typical amorphous carbon structure in the composite. It should be mentioned that no diffraction signals from the Fe or Zn related species could be detected, implying the formation of FeCo alloys embedded in the N-doped carbon skeleton. For the Fe-CoS_2_/NC-3 sample, it was noticed that apart from the carbon related peak at 26°, all of the diffraction signals from the FeCo alloys had diminished, and the new peaks that appeared at 28.0°, 32.5°, 36.4°, 40.1°, 46.5°, 55.2°, 57.8°, 60.3°, and 63.1° can be readily attributed to the (111), (200), (211), (210), (220), (311), (222), (023), and (321) crystalline planes of cubic CoS_2_ (JCPDS No. 89-3056), respectively, suggesting the complete conversion of FeCo alloys into Fe-CoS_2_ nanoparticles. The weight percentage of C was approximately 30%, which was detected from the EDS (Figure S2 and Table S1). Figure 3b–f shows the XPS spectra of the Fe-CoS_2_/NC-3 sample. In the case of the Co 2p spectrum, as presented in Figure 3b, the four deconvoluted peaks at 781.9, 798.4, 778.9, and 794.1 eV were ascribed to Co 2p_3/2_ and Co 2p_1/2_ of Co^2+^, Co 2p_3/2_ and Co 2p_1/2_ of Co^3+^ [47], respectively, while the two peaks at 785.0 and 803.3 eV were ascribed to the satellites, suggesting the existence of mixed Co^2+^ and Co^3+^, possibly originating from the partial surface oxidation of the metal sulfide. As displayed in Figure 3c, the Fe 2p spectrum with two characteristic peaks at 714.4 and 721.1 eV implies the presence of Fe^3+^ for the Fe-CoS_2_/NC-3 sample [48,49]. Figure 3d shows the S 2p spectrum, and there were two major peaks located at 163.2 and 164.5 eV, attributed to S 2p_3/2_ and S 2p_1/2_ of the metal-sulfur bonds, respectively [50,51]. For the C 1s spectrum depicted in Figure 3e, there were three deconvoluted peaks at 284.7, 286.0, and 287.0 eV related to the C–C/C=C, C–N and C–S bonds [52], respectively. The C–S bond clearly confirms the presence of interactions between the Fe-CoS_2_ nanoparticles and the N-doped carbon skeleton. Considering the N 1s spectrum in Figure 3f, the three main fitted peaks of 399.0, 401.0 and 402.2 eV were commonly indexed to pyridinic N, pyrrolic N, and graphitic N [53], respectively, suggesting that N atoms are successfully doped into the carbon skeleton. It is known that the presence of graphitic N through heteroatom doping can effectively contribute more electrons to the π-conjugated system with improved electrical conductivity [54], while the existing pyridinic N and pyrrolic N atoms in the carbon material can boost the sodium storage reaction by providing more active sites for Na^+^ insertion [55].

It should be mentioned that the formation reaction of the Fe-CoS_2_/NC hybrid spheres can be tailored by changing the amount of introduced FeCl_3_. To address the essential influence of FeCl_3_ on the formation of Fe-CoS_2_/NC hybrid spheres, we carried out a series of control experiments. Basically, with the absence of FeCl_3_ in the starting materials, the as-prepared pristine ZnCo-PBA spheres possessing a smooth surface and average diameter of about 1 μm were chemically transformed into the bayberry-like CoS_2_/NC hybrid spheres with a decreased average size of *ca.* 800 nm, as presented in Figure S3. In comparison, by introducing different amounts (*x* = 0.1, 0.2, 0.3, and 0.4 mmol) of FeCl_3_ in the starting materials, the samples denoted as Fe-CoS_2_/NC-1, Fe-CoS_2_/NC-2, Fe-CoS_2_/NC-3, and Fe-CoS_2_/NC-4 hybrid spheres were prepared through the successive carbonization and subsequent sulfurization treatment of the FeZnCo-PBA-1, FeZnCo-PBA-2, FeZnCo-PBA-3 and FeZnCo-PBA-4 precursor spheres, respectively (Figure S4). From Figures S3 and S4 and Table S2, we can see that the average diameters of the FeZnCo-PBA spheres as well as the resulting Fe-CoS_2_/NC hybrid spheres could be continuously reduced by introducing a greater amount of FeCl_3_ in the reaction. We propose that the appearance of this phenomenon is possibly due to the restricted growth of FeZnCo-PBA spheres with the presence of more FeCl_3_ in the reaction due to the competitive behavior of transition metal ions. Meanwhile, the structural variation of the Fe-CoS_2_/NC samples was also studied upon the introduction of different amounts of FeCl_3_. As presented in Figure S5, it was observed that the diffraction peaks from the CoS_2_ phase gradually shifted toward lower diffraction angles with slightly decreased intensities, clearly indicative of the enlarged lattice distances of the samples induced by the incorporation of Fe-atoms with a larger atom radius in the CoS_2_ lattice matrix [56]. Moreover, the practical Fe contents (Fe/(Fe+Co)) in the final samples of Fe-CoS_2_/NC hybrid spheres were investigated by EDS measurement. As shown in Figure S6, with the increase in the amount of introduced FeCl_3_ from 0.1 to 0.2 and 0.3 to 0.4 mmol, the practical Fe contents in the samples were determined to be 0.5 at.%, 3.7 at.%, 11.4 at.%, and 19.1 at.%, respectively, which were much lower than the feeding Fe contents. The presence of carbon materials in these samples was also explored by Raman spectra. As we can see, the obviously observed D band and G band at 1336 and 1588 cm^−1^, respectively, as displayed in Figure S7, were closely associated with the defect-related disordered carbon and graphitic carbon, respectively. On the basis of the results in Figure S7, it was revealed that the Fe-CoS_2_/NC-3 hybrid spheres exhibited a relatively larger peak intensity ratio (I_D_/I_G_) of 0.72 than those of the CoS_2_/NC (0.67), Fe-CoS_2_/NC-1 (0.66), Fe-CoS_2_/NC-2 (0.65), and Fe-CoS_2_/NC-4 (0.60) hybrid spheres, reflecting the higher defect degree in the carbon structure of the Fe-CoS_2_/NC-3 sample [57]. In addition, the BET surface area and pore structure of the Fe-CoS_2_/NC-3 and CoS_2_/NC samples were investigated by testing the N_2_ adsorption–desorption isotherms, as displayed in Figure S8. The Fe-CoS_2_/NC-3 sample exhibited a specific area of 144.8 m^2^ g^−1^, slightly larger than that of the CoS_2_/NC sample (134.1 m^2^ g^−1^). Furthermore, the detailed pore distribution curves indicate that the Fe-CoS_2_/NC-3 sample possessed concentrated mesopores with a size distribution of 2–5 nm, which was quite different from the bimodal size distribution of about 2.6 nm and 8.7 nm for the CoS_2_/NC sample. This suggests that the large specific area of the electrode material with a suitable mesopore structure can effectively increase the favorable diffusion path of Na^+^ and accelerate the reaction kinetics for the boosted electrochemical properties [58].

**Figure 4:**
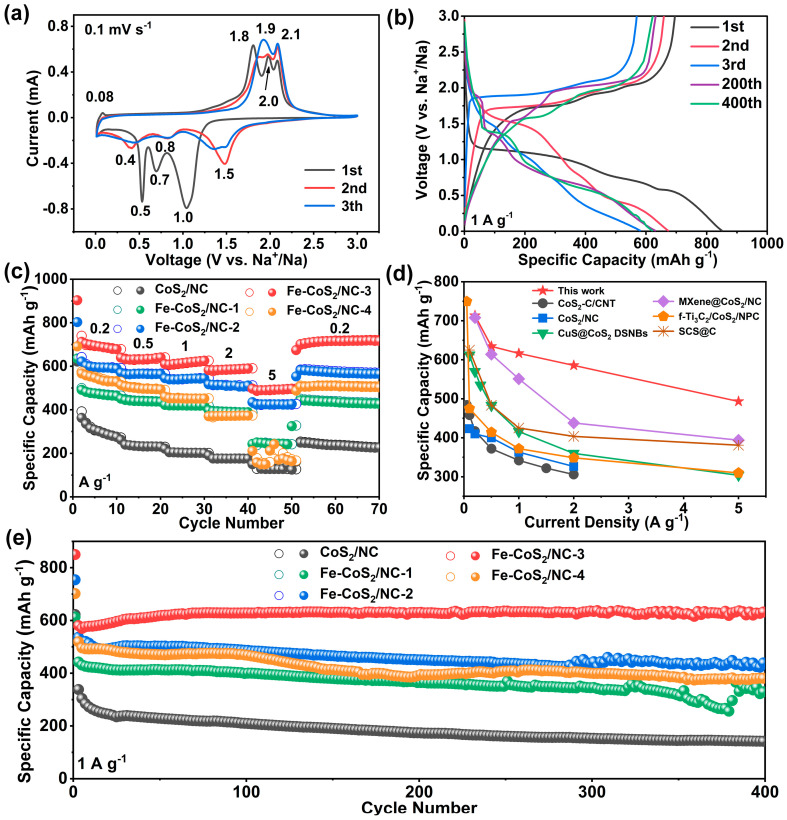
(**a**) CV curves of the Fe-CoS_2_/NC-3 electrode at 0.1 mV s^−1^. (**b**) GCD curves of the Fe-CoS_2_/NC-3 electrode at 1 A g^−1^, (**c**) comparison of the rate performance of Fe-CoS_2_/NC-3 with CoS_2_/NC, Fe-CoS_2_/NC-1, Fe-CoS_2_/NC-2, and Fe-CoS_2_/NC-4, (**d**) comparison of the rate capability with other CoS_2_-based electrodes as previously reported in the literature, (**e**) comparison of the long-term cycling performances of the electrodes at 1 A g^−1^.

The sodium storage properties of the optimal Fe-CoS_2_/NC-3 electrode was first evaluated in the assembled CR2025 coin-type half-cells. The specific capacities of the battery were calculated according to the total active mass of the Fe-CoS_2_/NC electrode. Figure 4a depicts the initial three cycles of the CV curves for the Fe-CoS_2_/NC-3 electrode recorded at 0.1 mV s^−1^ from 0.01 to 3.0 V. During the first sodiation process, the two dominant reduction peaks that appeared at around 1.0 and 0.7 V were assigned to the multi-step insertion of Na^+^ into Fe-CoS_2_ to form an intermediate product and then metallic Fe, Co, and Na_2_S, respectively, while the sharp reduction peak at 0.5 V was attributed to the generation of a solid electrolyte interphase (SEI) layer [59,60]. During the corresponding desodiation process, the three oxidation peaks at 1.8, 2.0, and 2.1 V were associated with the stepwise reverse conversion reaction between metallic Fe, Co, and Na_2_S to regenerate metal sulfides [61]. In the subsequent CV scans, it can be seen that the original reduction peaks moved to 1.5, 0.8, and 0.4 V, and the oxidation peaks appeared at new positions of 1.9 and 2.1 V, respectively, originating from the structural rearrangement and activation of the electrode materials [62]. Figure 4b depicts the GCD curves of the Fe-CoS_2_/NC-3 electrode measured at a current density of 1 A g^−1^ for the first three cycles and the 200th and 400th cycles. The obvious voltage platforms were consistent with the reduction and oxidation peaks in the CV profiles. The Fe-CoS_2_/NC-3 electrode delivered initial discharge and charge capacities of 850 and 696 mA h g^−1^, respectively, signifying an initial Coulombic efficiency (ICE) of 81.8%. Overpotential from the second cycle to the third cycle may indicate increased resistance and polarization due to SEI film formation [63]. During the charging and discharging process from the 200th to the 400th cycle, as the electrolyte fully infiltrates the micropores in the carbon sphere, the contact area between the active substance and the electrolyte is increased, thus achieving activation and reducing polarization [64,65]. The 200th and 400th cycles had similar voltage profiles, which showed the excellent reversibility of the electrodes for Na^+^ storage [66]. The rate performance of the Fe-CoS_2_/NC-3 electrode is presented in Figure 4c, compared with those of the CoS_2_/NC, Fe-CoS_2_/NC-1, Fe-CoS_2_/NC-2, and Fe-CoS_2_/NC-4 electrodes. Remarkably, the Fe-CoS_2_/NC-3 electrode exhibited high reversible capacities of 713, 635, 616, 586, and 493 mA h g^−1^ at varied current densities from 0.2 to 0.5, 1, 2, and 5 A g^−1^, respectively. Moreover, when the current density was reset to 0.2 A g^−1^, the reversible capacity of the Fe-CoS_2_/NC-3 electrode could still retain a high value of 717 mA h g^−1^ after 20 cycles with an approximately 84.3% retention. It is worth noting that during the whole current density range, the Fe-CoS_2_/NC-3 electrode displayed a much better rate capability than those of the CoS_2_/NC, Fe-CoS_2_/NC-1, Fe-CoS_2_/NC-2, and Fe-CoS_2_/NC-4 electrodes, especially at high rates. Notably, the reversible capacity of the Fe-CoS_2_/NC-3 electrode at 0.2 A g^−1^ and 5 A g^−1^ was about 2.1 and 3.6 times higher than those for CoS_2_/NC, respectively. Additionally, the superior rate capability of the Fe-CoS_2_/NC-3 electrode can be further illustrated by a comparison with that of some recently reported CoS_2_-based anodes such as a CoS_2_/C micropolyhedron composite entangled in a carbon-nanotube base network (CoS_2_-C/CNT) [44], CoS_2_ nanoparticles embedded in N-doped carbon nanosheets (CoS_2_/CN) [67], CuS@CoS_2_ double shelled nanoboxes (CuS@CoS_2_ DSNBs) [20], CoS_2_ nanoparticles embedded in N-doped carbon grown on MXene nanosheets (MXene@CoS_2_/CN) [29], Ti_3_C_2_ MXene/CoS_2_@N-doped porous carbon (f-Ti_3_C_4_/CoS_2_/NPC) [68], and SnS_2_-CoS_2_@C core-shell nanocubes (SCS@C) [69], as shown in Figure 4d. The long-term cycling stabilities of the Fe-CoS_2_/NC-3, CoS_2_/NC, Fe-CoS_2_/NC-1, Fe-CoS_2_/NC-2, and Fe-CoS_2_/NC-4 electrodes were also evaluated at 1 A g^−1^, as shown in Figure 4e. For the Fe-CoS_2_/NC-3 electrode, the capacity decayed from 850 mA h g^−1^ to 673 mA h g^−1^ at the second cycle, and gradually stabilized at 621 mA h g^−1^ over 400 cycles with a CE approaching 100%, showing a retention of 92.2% to the second cycle, suggesting the good stability of the Fe-CoS_2_/NC-3 electrode during the cycling process. In contrast, the CoS_2_/NC, Fe-CoS_2_/NC-1, Fe-CoS_2_/NC-2, and Fe-CoS_2_/NC-4 electrodes displayed relatively poor cycling stabilities, which only retained lower values of 313, 352, 432, and 382 mA h g^−1^ over 400 cycles with a retention of 83.9%, 74.3%, 74.1, and 67.5% compared to the second cycle, respectively. In addition, Figure S9 shows that the Fe-CoS_2_/C-3 electrode still presented a specific capacity as high as 665 mA h g^−1^ at 1 A g^−1^ over 900 cycles with a retention of 78.2%, further confirming the good long-term cycling stability of the Fe-CoS_2_/NC-3 electrode.

**Figure 5:**
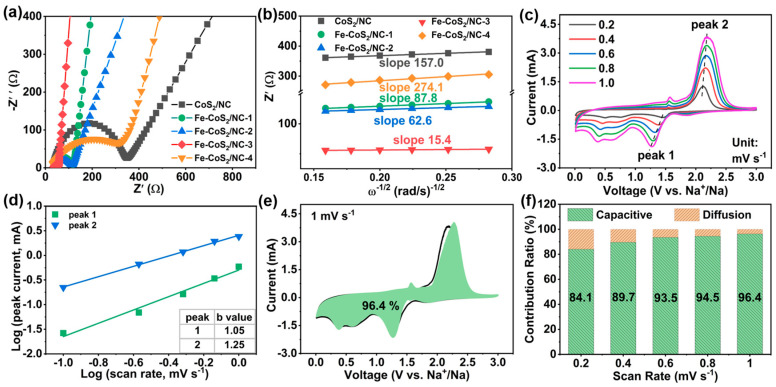
(**a**) Nyquist plots and (**b**) linear fitting of *Z*^′^ *versus ω*^−1/2^ of the Fe-CoS_2_/NC-3, CoS_2_/NC, Fe-CoS_2_/NC-1, Fe-CoS_2_/NC-2, and Fe-CoS_2_/NC-4 electrodes, for these electrodes, (**c**) the CV curves at different scan rates, (**d**) linear relationship between log(*i*) and log(*v*) for selected peaks of CV curves, (**e**) the capacitive contribution at 1.0 mV s^−1^ marked by the colored region, and (**f**) contribution ratio of the capacitive and diffusion-controlled capacities at different scan rates for the Fe-CoS_2_/NC-3 electrode.

In order to better understand the superior rate capability and cycling stability of the Fe-CoS_2_/NC-3 electrode, the detailed electrochemical reaction kinetics was investigated through EIS and CV measurements. As presented in Figure 5a, all of the Nyquist plots contained a depressed semicircle in the high-frequency region corresponding to the charge transfer resistance (*R_ct_*), and an oblique line in the low-frequency region related to the Warburg impedance (*Z_w_*) of Na^+^ diffusion in the electrode [70]. As expected, the Fe-CoS_2_/NC-3 electrode revealed a much smaller *R_ct_* of 18.7 Ω compared with those of CoS_2_/NC (308.7 Ω), Fe-CoS_2_/NC-1 (83.1 Ω), Fe-CoS_2_/NC-2 (78.4 Ω), and Fe-CoS_2_/NC-4 (351.4 Ω), respectively, verifying the enhanced charge transfer ability of the Fe-CoS_2_/NC-3 electrode. Meanwhile, as one of the key parameters to measure the reaction dynamics, the Na^+^ diffusion coefficient ($D_{{\mathrm{Na}}^{+}}$) can be estimated from the low-frequency region of the Nyquist plots according to the following equation [71]:

(1) $$D_{{\mathrm{Na}}^{+}}=0.5R^{2}T^{2}/S^{2}n^{4}F^{4}C^{2}\sigma^{2}$$

where *T* is the absolute temperature; *R* is the gas constant; *S* is the contact area of the electrode; *n* is the number of reaction transition electrons; *C* is the Na^+^ concentration; F is the Faraday constant; and *σ* is the Warburg factor. Basically, the value of the Warburg factor *σ* can be derived from the linearly fitted slope by plotting *Z′ versus ω*^−1/2^, as shown in Figure 5b. Therefore, the values of $D_{{\mathrm{Na}}^{+}}$ for the Fe-CoS_2_/NC-3 electrode can be calculated to be 2.5 × 10^−12^ cm^2^ s^−1^, which is about one order of magnitude higher than those of CoS_2_/NC (2.5 × 10^−13^ cm^2^ s^−1^), Fe-CoS_2_/NC-1 (4.4 × 10^−13^ cm^2^ s^−1^), Fe-CoS_2_/NC-2 (6.2 × 10^−13^ cm^2^ s^−1^), and Fe-CoS_2_/NC-4 (1.4 × 10^−13^ cm^2^ s^−1^), respectively, confirming that the Fe-CoS_2_/NC-3 electrode possessed a much faster Na^+^ diffusion rate. The kinetics property was further studied by CV measurements at different scan rates ranging from 0.2 to 1.0 mV s^−1^. As shown in Figure 5c, the CV profiles of the Fe-CoS_2_/NC-3 electrode maintained similar shapes at all scan rates, reflecting the low polarization of the electrode. As we know, the capacitive and diffusion behaviors of the electrode can be evaluated from the relationship between the peak current (*i*) and scan rate (*v*) according to the following equation [72]:

(2) $$i=a\nu^{b}$$

The *b* value can be derived from the slope by plotting log(*i*) *versus* log(*v*), and the *b* value of 0.5 or 1 indicates that the diffusion or capacitive contribution leads to the electrochemical reaction. As displayed in Figure 5d, the *b* values for the two selected peaks were determined to be 1.05 and 1.25, suggesting that the capacitive behavior dominates the sodium storage process. In addition, the detailed contribution proportion from the capacitive (*k*_1_*v*) and diffusion (*k*_2_*v*^1/2^) behaviors can be further quantified based on the following equation [73]:

(3) $$i=k_{1}v+k_{2}v^{1/2}$$

where *k*_1_ and *k*_2_ are two constants derived from the linear relationship by plotting *iv*^1/2^ *versus v*^1/2^ at a fixed voltage. Figure 5e shows the capacitive contribution of 96.4% for the Fe-CoS_2_/NC-3 electrode, as indicated by the colored area under 1 mV s^−1^. With the scan rate increasing from 0.2 to 1.0 mV s^−1^ (Figure 5f), the proportion of the capacitive contribution gradually increased from 84.1% to 96.4%. On the basis of the above results, we can draw a conclusion that the boosted electrical and ion transport ability with favorable reaction kinetics can well account for the outstanding rate capability and cycling stability of the Fe-CoS_2_/NC-3 electrode.

**Figure 6:**
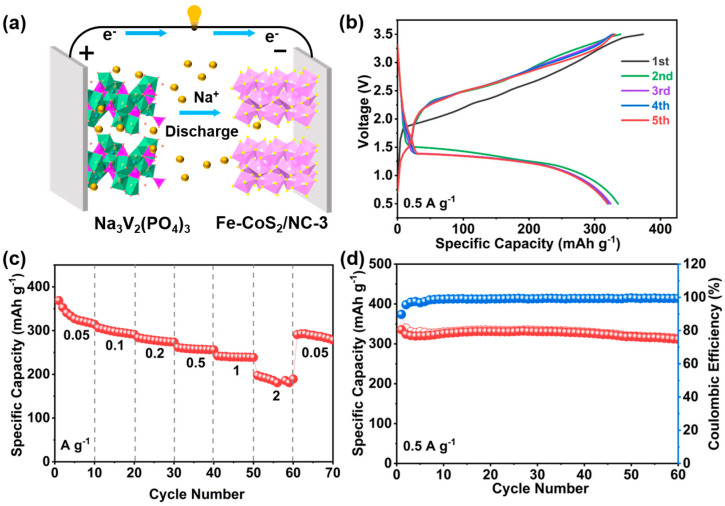
Electrochemical performance of the Fe-CoS_2_/NC-3//NVP full SIBs: (**a**) schematic diagram of the full battery, (**b**) initial five cycles of GCD curves at 0.5 A g^−1^, (**c**) rate capability, and (**d**) cycling stability at 0.5 A g^−1^.

Motivated by the superior sodium ion storage properties of the Fe-CoS_2_/NC-3 hybrid spheres in half-cells, the practical application prospect of the Fe-CoS_2_/NC-3 electrode was further investigated through the assembly of full devices by coupling with the commercial Na_3_V_2_(PO_4_)_3_ (NVP) as the cathode, as schematically indicated in Figure 6a. Figure 6b displays the GCD curves of the Fe-CoS_2_/NC-3//NVP device of initial five cycles, as measured at 0.5 A g^−1^ in the voltage window between 0.5 and 3.5 V. It revealed that the initial charge and discharge capacities were 373 and 335 mA h g^−1^, as derived on the basis of the active mass of anode materials [74], showing a CE of 89.8%. Figure 6c presents the rate performance of the full battery, which delivered an average capacity of 356, 298, 278, 258, 240, and 184 mA h g^−1^ at current densities of 0.05, 0.1, 0.2, 0.5, 1, and 2 A g^−1^, respectively. Furthermore, the capacity could quickly recover to a high value of 302 mA h g^−1^ when the current density changed back to 0.05 A g^−1^, suggesting the reasonably good rate performance of the full battery. The commercial Na_3_V_2_(PO_4_)_3_ was tested in the half cells first, and the test revealed that the NVP cathode electrode had an initial capacity of 103 mA h g^−1^, showing a specific capacity as high as 93 mA h g^−1^ at 0.2 A g^−1^ over 100 cycles with a retention of 90.3% to the second cycle (Figure S10). Figure 6d shows the cycling performance of the Fe-CoS_2_/NC-3//NVP device tested at 0.5 A g^−1^. The capacity of the device was still maintained at 313 mA h g^−1^ over 60 cycles with a retention of 83.9%. Additionally, the energy density and power density of the Fe-CoS_2_/NC-3//NVP full SIBs in this study were compared with the previously reported metal sulfide-NVP full SIBs [73,75,76,77,78,79,80], as summarized in Table S3, further demonstrating the certain application potential of the Fe-CoS_2_/NC-3//NVP full battery.

## 4. Conclusions

In this work, we proposed a new approach to synthesize PBA-derived bayberry-like Fe-CoS_2_/NC hybrid spheres comprising Fe-doped CoS_2_ nanoparticle confined N-doped carbon spheres. The electron and ion transport ability of the composite can be greatly improved by the exterior N-doped carbon spheres with rich porosity originating from the thermal decomposition of the introduced Pluronic F127 and the evaporation of the as-formed Zn species at high temperature, while the incorporated Fe^3+^ ions in CoS_2_ can provide more active sites and contribute to the increased capacity. By virtue of these advantages, the optimal Fe-CoS_2_/NC-3 anode showed a high sodium storage capacity with a superior cycling stability (621 mA h g^−1^ after 400 cycles at 1 A g^−1^) and improved rate capability (493 mA h g^−1^ at 5 A g^−1^). We suggest that this work provides a novel synthetic route for the construction of high performance metal sulfide-based anode materials applied in SIBs.

## Data Availability

Not applicable.

## Supplementary Materials

The following supporting information can be downloaded at: https://www.mdpi.com/article/10.3390/polym15061496/s1, Figure S1: XPS survey spectrum of the Fe-CoS_2_/NC-3; Figure S2: EDS spectrum of the Fe-CoS_2_/NC-3 sample; Figure S3: SEM images of the (a) ZnCo-PBA precursor spheres, and (b) CoS_2_/NC spheres; Figure S4: SEM images of the (a) FeZnCo-PBA-1, (b) FeZnCo-PBA-2, (c) FeZnCo-PBA-3, (d) FeZnCo-PBA-4 precursor spheres, (e) Fe-CoS_2_/NC-1, (f) Fe-CoS_2_/NC-2, (g) Fe-CoS_2_/NC-3, and (h) Fe-CoS_2_/NC-4 hybrid spheres; Figure S5: (a) XRD patterns of the CoS_2_/NC, Fe-CoS_2_/NC-1, Fe-CoS_2_/NC-2, Fe-CoS_2_/NC-3, Fe-CoS_2_/NC-4 spheres in the range of 10–80°, and (b) zoom view of (a) in the range of 30–46°; Figure S6: The practical Fe contents in the products and the feeding Fe contents in the starting materials of Fe-CoS_2_/NC-1, Fe-CoS_2_/NC-2, Fe-CoS_2_/NC-3, and Fe-CoS_2_/NC-4; Figure S7: Raman spectra of CoS_2_/NC, Fe-CoS_2_/NC-1, Fe-CoS_2_/NC-2, Fe-CoS_2_/NC-3, and Fe-CoS_2_/NC-4; Figure S8: (a) N_2_ adsorption–desorption isotherms curves of CoS_2_/NC and Fe-CoS_2_/NC-3, and (b) the pore size distributions of the CoS_2_/NC and Fe-CoS_2_/NC-3; Figure S9: Long-term cycling performance of the Fe-CoS_2_/NC-3 electrode; Figure S10: Cycling performance of NVP at current density of 0.2 A g^−1^; Table S1: The element contents in Fe-CoS_2_/NC-3 measured by EDS measurement; Table S2: The average diameters of the precursors and obtained samples derived from the reactions with different introduced amounts of FeCl_3_; Table S3: Comparison of electrochemical performance of Fe-CoS_2_/NC-3//NVP full SIBs in this work with previously reported metal sulfide-NVP full SIBs.
