# Supplementary material for: Prussian Blue Analogue-Derived Fe-Doped CoS2 Nanoparticles Confined in Bayberry-like N-Doped Carbon Spheres as Anodes for Sodium-Ion Batteries
Source: Polymers (Basel). 2023 Mar 17;15(6):1496. doi: 10.3390/polym15061496 (PMC10054790; doi:10.3390/polym15061496)
Supplement: Supplementary file 1 [file polymers-15-01496-s001.zip › polymers-2231264-SI.pdf]

Supplementary Material for

Prussian Blue Analogue-Derived Fe-Doped CoS_2 Nanoparticles Confined in Bayberry-Like N-Doped Carbon Spheres as Anodes for Sodium-Ion Batteries

Jiajia Hu, Cheng Liu, Chen Cai, Qianqian Sun, Mixue Lu, Zhujun Yao, and Yefeng Yang*

School of Materials Science and Engineering, Zhejiang Sci-Tech University, Hangzhou 310018, China

* *Correspondence: yangyf@zstu.edu.cn; Tel.: +86-571 8684 5569*

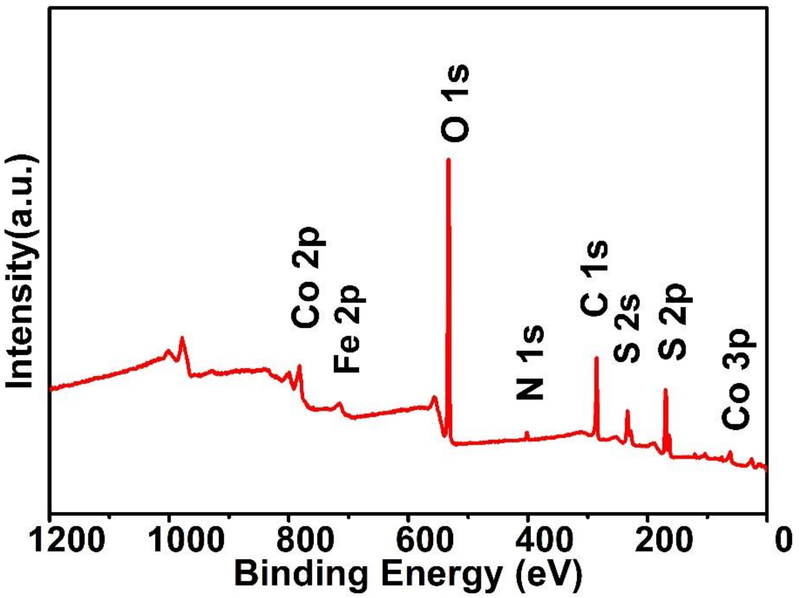

Figure S1. XPS survey spectrum of the Fe-CoS_2/NC-3.

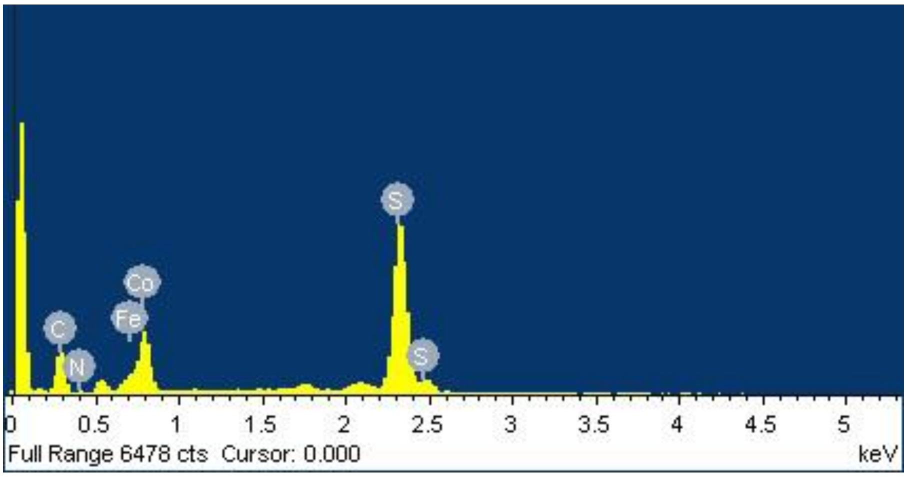

Figure S2. EDS spectrum of the Fe-CoS_2/NC-3 sample.

Table S1. The element contents in Fe-CoS_2/NC-3 measured by EDS measurement.

Element	Co	Fe	S	C	N	Total
Contents (wt%)	30.0	3.7	33.0	31.3	2.1	100.0

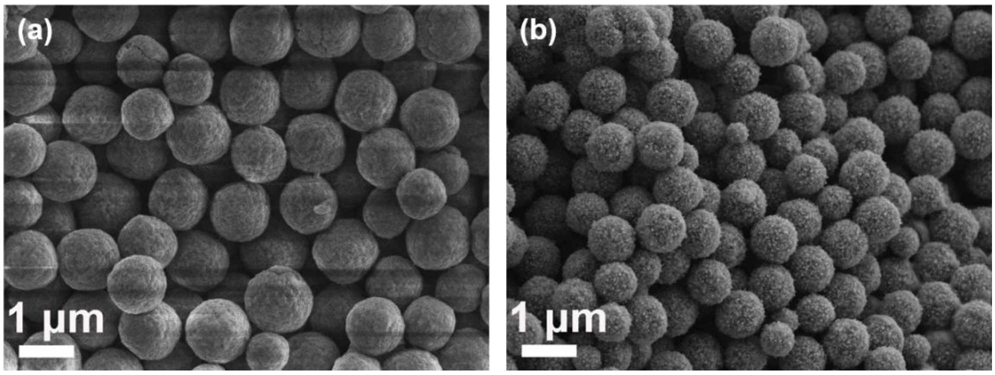

Figure S3. SEM images of (a) ZnCo-PBA precursor spheres, and (b) CoS_2/NC spheres.

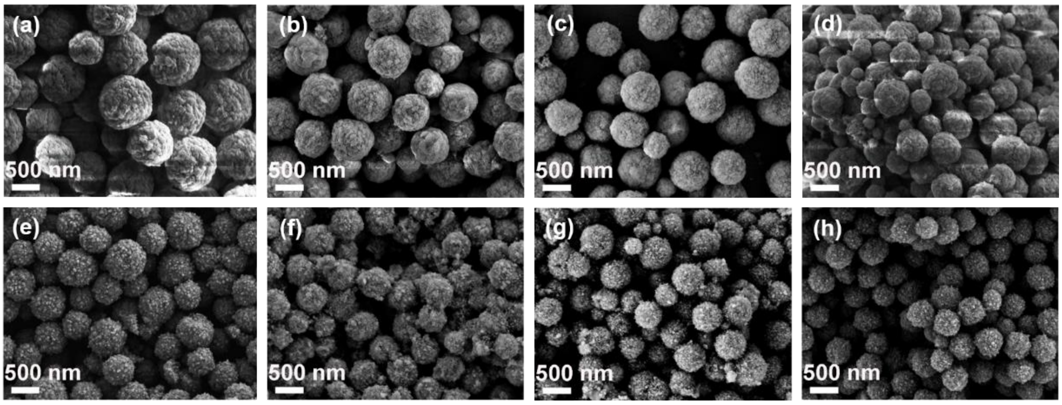

Figure S4. SEM images of (a) FeZnCo-PBA-1, (b) FeZnCo-PBA-2, (c) FeZnCo-PBA-3, (d) FeZnCo-PBA-4 precursor spheres, (e) Fe-CoS_2/NC-1, (f) Fe-CoS_2/NC-2, (g) Fe-CoS_2/NC-3, and (h) Fe-CoS_2/NC-4 hybrid spheres.

Table S2. The average diameters of precursors and obtained samples derived from the reactions with different introduced amounts of $FeCl_3$.

Precursors	Average diameter	Samples	Average diameter
ZnCo-PBA	1000 nm	CoS_2/NC	810 nm
FeZnCo-PBA-1	723 nm	Fe-CoS_2/NC-1	482 nm
FeZnCo-PBA-2	583 nm	Fe-CoS_2/NC-2	478 nm
FeZnCo-PBA-3	572 nm	Fe-CoS_2/NC-3	450 nm
FeZnCo-PBA-4	496 nm	Fe-CoS_2/NC-4	408 nm

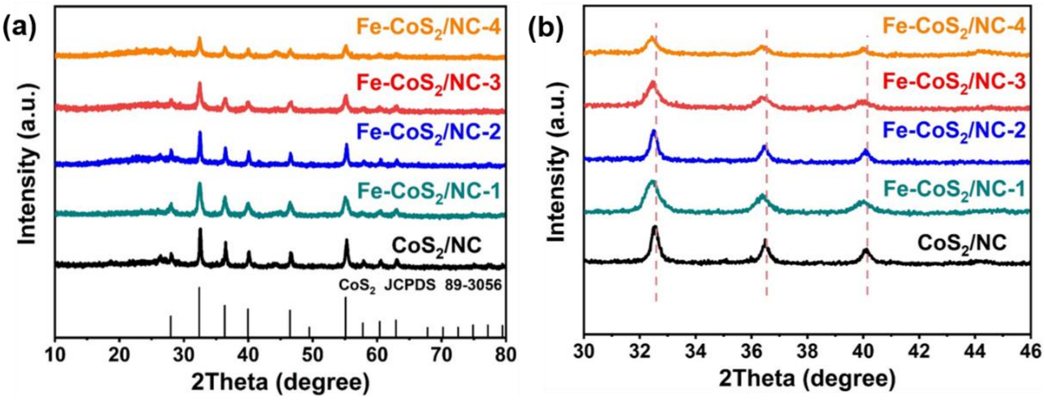

Figure S5. (a) XRD patterns of CoS_2/NC, Fe-CoS_2/NC-1, Fe-CoS_2/NC-2, Fe-CoS_2/NC-3, and Fe-CoS_2/NC-4 spheres in the range of 10-80°, and (b) zoom view of (a) in the range of 30-46°.

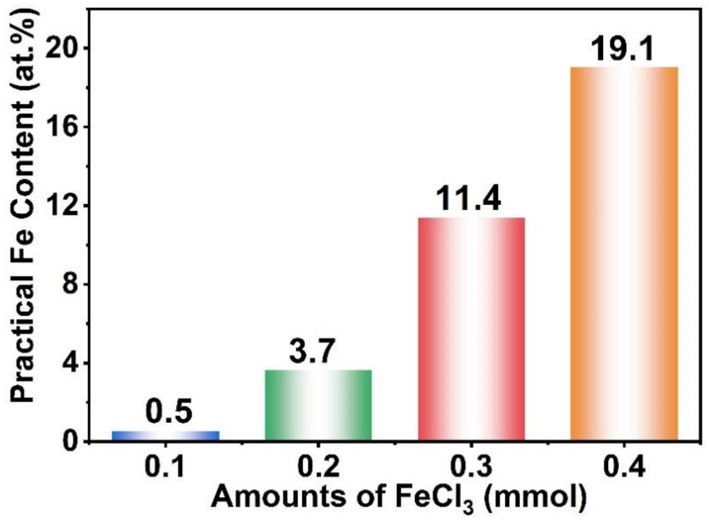

Figure S6. The practical Fe contents in products and the feeding Fe contents in the starting materials of Fe-CoS_2/NC-1, Fe-CoS_2/NC-2, Fe-CoS_2/NC-3, and Fe-CoS_2/NC-4.

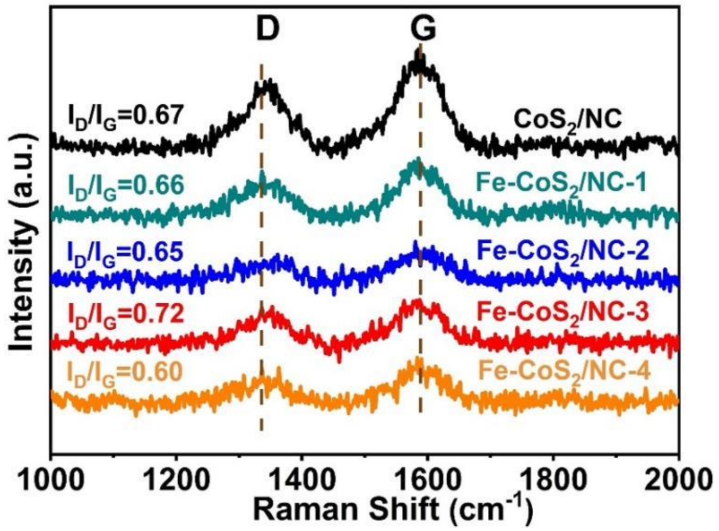

Figure S7. Raman spectra of CoS_2/NC, Fe-CoS_2/NC-1, Fe-CoS_2/NC-2, Fe-CoS_2/NC-3, and Fe-CoS_2/NC-4.

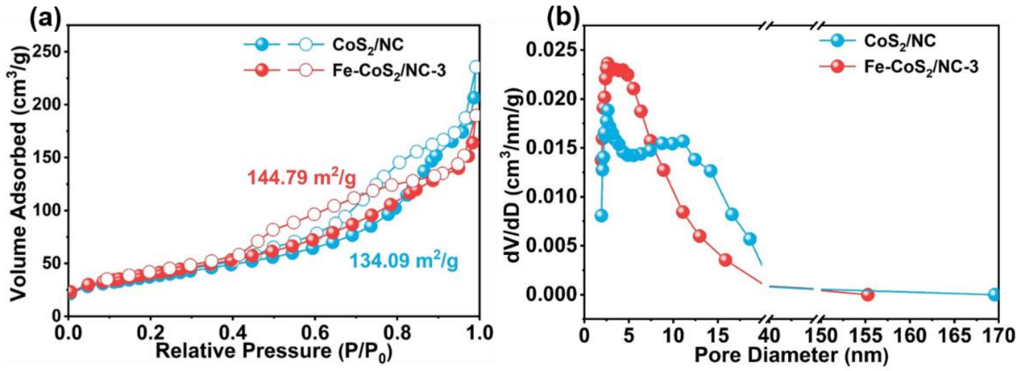

Figure S8. (a) N_2 adsorption-desorption isotherms curves of CoS_2/NC and Fe-CoS_2/NC-3, and (b) pore size distributions of the CoS_2/NC and Fe-CoS_2/NC-3.

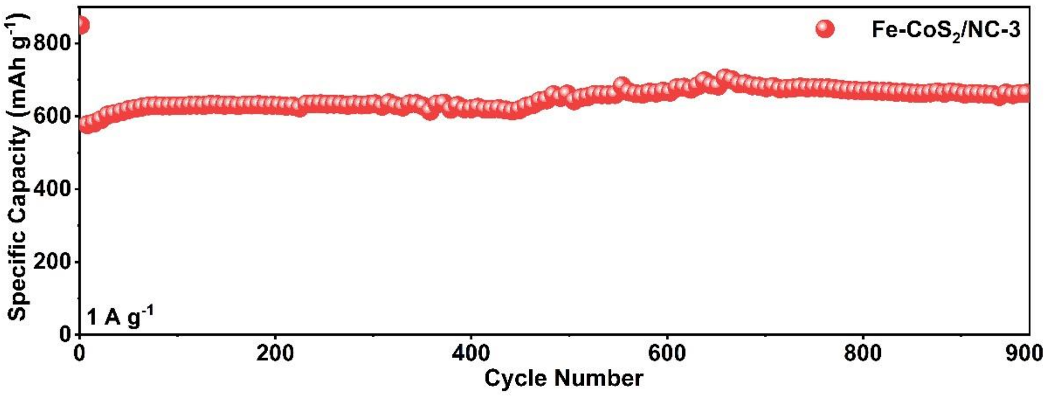

Figure S9. Long-term cycling performance of the Fe-CoS_2/NC-3 electrode.

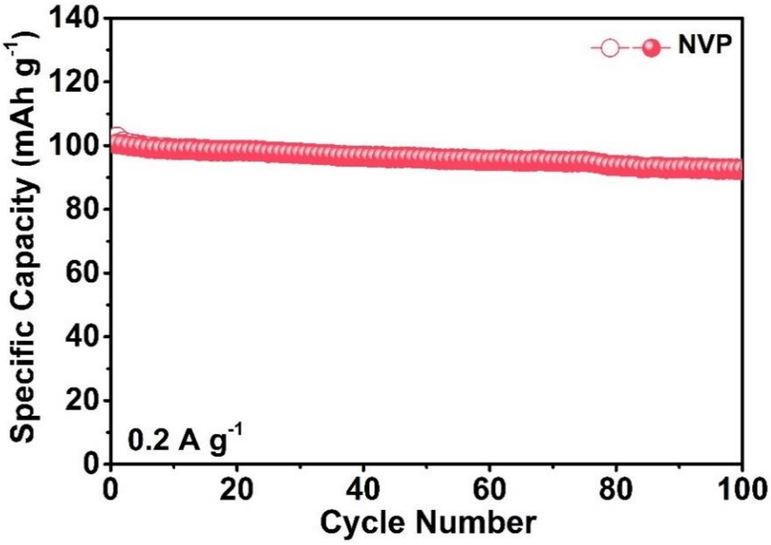

Figure S10. Cycling performance of NVP at current density of 0.2 A g^{-1}.

Table S3. Comparison of electrochemical performance of Fe-CoS_2/NC-3//NVP full SIBs in this work with previously reported metal sulfide-NVP full SIBs.

Anode	Cathode	Capacity/current density/cycles (mAh g^{-1}/A g^{-1}/n)	Energy density/current density (Wh kg^{-1}/A g^{-1})	Power density (W kg^{-1})	Ref.
Fe-CoS_2/NC-3	NVP	313/0.5/60	102/0.2	74	This work
SnS/Fe_2O_3-G	NVP	51/1/200	141.8/0.2	60	[75]
FeS@NCG	NVP@C	308/0.6/150	218/0.6	–	[76]
FeS_2@G@CNF	NVP@C	102/1C/100	169/1C	88	[77]
V_3S_4@CNF	NVP/C	365/0.1/20	400/0.1	–	[78]
$Ti_3C_2T_x$/FeS_2	NVP	365/3/200	80/5	–	[79]
Cu-SnS_2@NC	NVP	223/0.5/50	233	240	[80]
NiS_2@SnS_2	NVP	628/0.5/150	274	52.1	[73]
